# Ammonium piperidine-1-carbodithio­ate

**DOI:** 10.1107/S1600536811008129

**Published:** 2011-03-15

**Authors:** Ana C. Mafud, Maria T. P. Gambardella

**Affiliations:** a Instituto de Química de São Carlos, Universidade de São Paulo, Av. Trabalhador Sãocarlense 400, Caixa Postal 780, 13560-970 São Carlos, SP, Brazil

## Abstract

The title compound, NH_4_^+^·C_6_H_10_NS_2_^−^, is composed of an ammonium cation and a piperidine-1-carbodithio­ate anion which exhibits positional disorder. The atoms of the ring have a structural disorder and they are divided into two sites, with occupancy factors of 0.584 and 0.426.. In the crystal, the cation and anion are linked by N—H⋯S hydrogen bonds to form an infinite two-dimensional network.

## Related literature

For the crystal structures of similar compounds, see: Wahlberg (1979 ▶, 1980 ▶, 1981 ▶).
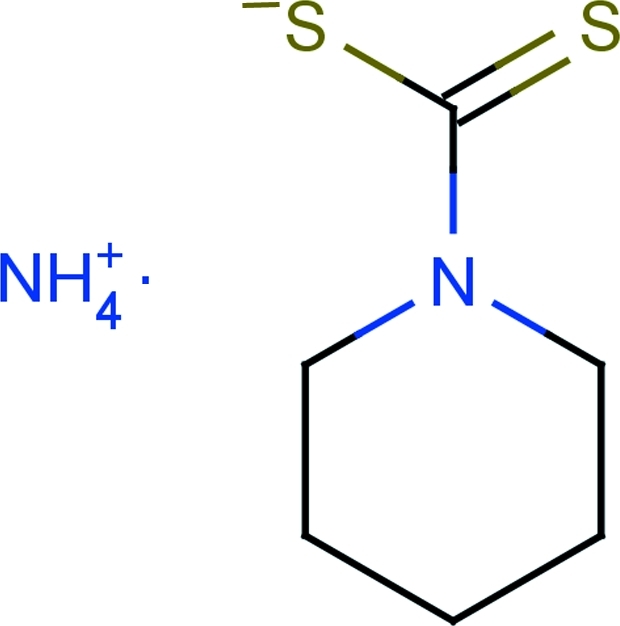


## Experimental

### 

#### Crystal data

NH_4_^+^·C_6_H_10_NS_2_^−^
*M* *_r_* = 178.31
Monoclinic, 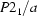

*a* = 8.8812 (9) Å
*b* = 9.0025 (9) Å
*c* = 11.8995 (5) Å
β = 104.318 (5)°
*V* = 921.85 (14) Å^3^
*Z* = 4
Mo *K*α radiation
μ = 0.51 mm^−1^
*T* = 290 K
0.40 × 0.35 × 0.13 mm

#### Data collection

Enraf–Nonius TurboCAD-4 diffractometer
Absorption correction: ψ scan (North *et al.*, 1968 ▶) *T*_min_ = 0.582, *T*_max_ = 0.936
2847 measured reflections
2684 independent reflections
2093 reflections with *I* > 2σ(*I*)
*R*_int_ = 0.015
3 standard reflections every 120 min  intensity decay: 5%

#### Refinement

*R*[*F* ^2^ > 2σ(*F* ^2^)] = 0.042
*wR*(*F* ^2^) = 0.123
*S* = 1.06
2684 reflections
153 parameters
H atoms treated by a mixture of independent and constrained refinement
Δρ_max_ = 0.57 e Å^−3^
Δρ_min_ = −0.29 e Å^−3^

### 

Data collection: *CAD-4 EXPRESS* (Enraf–Nonius, 1994 ▶); cell refinement: *CAD-4 EXPRESS*; data reduction: *XCAD4* (Harms & Wocadlo, 1995 ▶); program(s) used to solve structure: *SHELXS97* (Sheldrick, 2008 ▶); program(s) used to refine structure: *SHELXL97* (Sheldrick, 2008 ▶); molecular graphics: *ORTEP-3 for Windows* (Farrugia, 1997 ▶); software used to prepare material for publication: *WinGX* (Farrugia, 1999 ▶).

## Supplementary Material

Crystal structure: contains datablocks global, I. DOI: 10.1107/S1600536811008129/su2257sup1.cif

Structure factors: contains datablocks I. DOI: 10.1107/S1600536811008129/su2257Isup2.hkl

Additional supplementary materials:  crystallographic information; 3D view; checkCIF report

## Figures and Tables

**Table 1:** Hydrogen-bond geometry (Å, °)

*D*—H⋯*A*	*D*—H	H⋯*A*	*D*⋯*A*	*D*—H⋯*A*
N1—H1⋯S2^i^	0.78 (3)	2.64 (3)	3.4029 (19)	167 (2)
N1—H2⋯S1	0.89 (3)	2.49 (3)	3.3565 (19)	164 (2)
N1—H3⋯S1^ii^	0.93 (3)	2.51 (3)	3.3967 (19)	159 (2)
N1—H4⋯S2^iii^	0.89 (3)	2.48 (3)	3.3632 (19)	170 (3)

Symmetry codes: (i) 
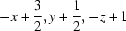
; (ii) 
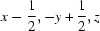
; (iii) 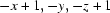.

**Fig. 1.**
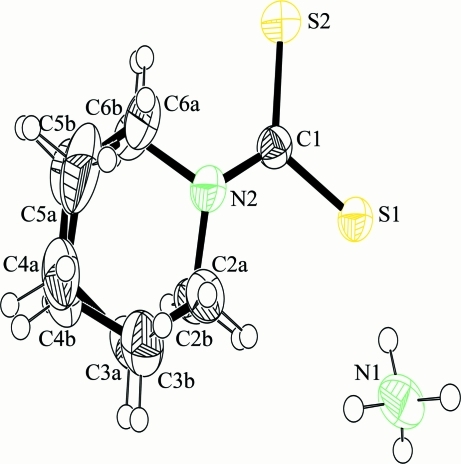
A view of the molecular structure of the title compound, with displacement ellipsoids drawn at the 50% probability level.

**Fig. 2.**
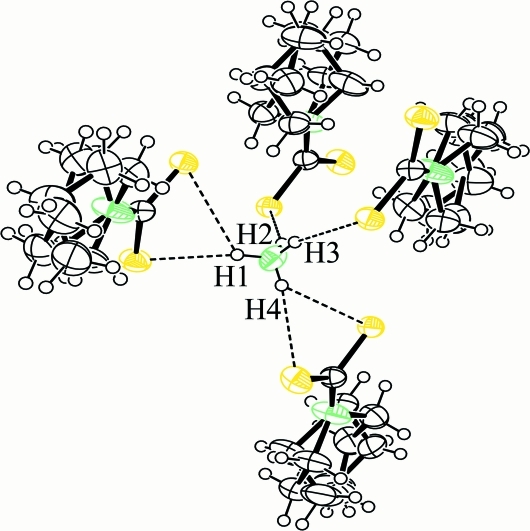
A view of the ammonium cation surrounded by four piperidinedithiocarbamate anions that are linked via N—H···S hydrogen bonds (see Table 1 for details). The N—H···S hydrogen bonds are shown as dotted lines.

**Fig. 3.**
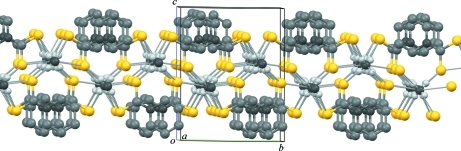
A view along the a-axis of the crystal packing of the title compound. The N—H···S hydrogen bonds are shown as dotted lines [The minor disordered fraction of the piperidine ring and the C-bound H-atoms have been omitted for clarity; colour code: S yellow; N black; C grey; H off-white].

## supplementary crystallographic information

### Comment

The title compound is compossed of an ammonium cation and a piperidinedithiocarbamate anion. The crystal structures of similar compounds, for example pyrrolidinium 1-pyrrolidinecarbodithioate (Wahlberg, 1979), and β and α piperidinium 1-piperidinecarbodithionate (Wahlberg, 1980, 1981), have been reported.

The molecular structure of the title compound (Fig. 1) is built up of an ammonium cation and a disordered piperidinedithiocarbamate anion. The carbon atoms (C2-C6) are disordered, occupying two positions (A/B) with occupancies of 0.584 (8)/0.416 (8)

In the crystal the cation is linked to four piperidinedithiocarbamate anions via N-H···S hydrogen bonds (Table 1 and Fig. 2). These interactions lead to the formaion of an infinite two-dimensional network (Fig. 3), propagating in (001).

### Experimental

The title compound was prepared by slow addition of 0.1 mol of CS_2_ to a cold solution containing 0.2 mol of ammonia and 0.2 mol of piperidine dissolved in 30 ml of ethanol-water 1:1 (*v*/*v*) medium. The mixture was kept in an ice bath during the reaction. The solid obtained was recrystallized from ethanol-water 1:1 (*v*/*v*) and dried in a vacuum oven at 323 K for 8 h. Colourless single crystals, suitable for X-ray diffraction analysis, were obtained. On heating they sublimed and decomposed.

### Refinement

The H-atom positions of the ammonium cation were located in a difference Fouier map and were freely refined: N-H = 0.78 (3) - 0.93 (3) Å. The C-bound H-atoms of the anion were included in calculated positions and treated as riding atoms: C-H = 0.97 Å, with U_iso_(H) = 1.2U_eq_(parent C-atom).

### Crystal data

**Table:**

NH_4_^+^·C_6_H_10_NS_2_^−^	*F*(000) = 384
*M**_r_* = 178.31	*D*_x_ = 1.285 Mg m^−^^3^
Monoclinic, *P*2_1_/*a*	Mo *K*α radiation, λ = 0.71073 Å
Hall symbol: -P 2yab	Cell parameters from 14 reflections
*a* = 8.8812 (9) Å	θ = 12.0–18.1°
*b* = 9.0025 (9) Å	µ = 0.51 mm^−^^1^
*c* = 11.8995 (5) Å	*T* = 290 K
β = 104.318 (5)°	Prism, colourless
*V* = 921.85 (14) Å^3^	0.40 × 0.35 × 0.13 mm
*Z* = 4	

### Data collection

**Table:**

Enraf–Nonius TurboCAD-4 diffractometer	2093 reflections with *I* > 2σ(*I*)
Radiation source: fine-focus sealed tube	*R*_int_ = 0.015
graphite	θ_max_ = 30.0°, θ_min_ = 2.9°
non–profiled ω/2θ scans	*h* = 0→12
Absorption correction: ψ scan (North *et al.*, 1968)	*k* = −12→0
*T*_min_ = 0.582, *T*_max_ = 0.936	*l* = −16→16
2847 measured reflections	3 standard reflections every 120 min
2684 independent reflections	intensity decay: 5%

### Refinement

**Table:**

Refinement on *F*^2^	0 restraints
Least-squares matrix: full	H atoms treated by a mixture of independent and constrained refinement
*R*[*F*^2^ > 2σ(*F*^2^)] = 0.042	*w* = 1/[σ^2^(*F*_o_^2^) + (0.075*P*)^2^ + 0.1152*P*] where *P* = (*F*_o_^2^ + 2*F*_c_^2^)/3
*wR*(*F*^2^) = 0.123	(Δ/σ)_max_ = 0.022
*S* = 1.06	Δρ_max_ = 0.57 e Å^−^^3^
2684 reflections	Δρ_min_ = −0.29 e Å^−^^3^
153 parameters	

### Special details

**Table:**

Geometry. Bond distances, angles etc. have been calculated using the rounded fractional coordinates. All su's are estimated from the variances of the (full) variance-covariance matrix. The cell esds are taken into account in the estimation of distances, angles and torsion angles

### Fractional atomic coordinates and isotropic or equivalent isotropic displacement parameters (Å^2^)

**Table:**

	*x*	*y*	*z*	*U*_iso_*/*U*_eq_	Occ. (<1)
S1	0.77666 (5)	0.04141 (4)	0.55813 (3)	0.0404 (1)	
S2	0.83657 (6)	−0.11212 (5)	0.78409 (4)	0.0531 (1)	
N2	0.8144 (3)	0.17995 (18)	0.76181 (14)	0.0669 (6)	
C1	0.80997 (19)	0.05002 (17)	0.70767 (14)	0.0391 (4)	
C2A	0.7452 (6)	0.3162 (4)	0.6942 (3)	0.0549 (13)	0.584 (8)
C3A	0.8316 (9)	0.4502 (5)	0.7551 (5)	0.0625 (15)	0.584 (8)
C4A	0.7868 (18)	0.4519 (18)	0.8807 (15)	0.077 (4)	0.584 (8)
C5A	0.8614 (7)	0.3215 (5)	0.9445 (4)	0.0629 (15)	0.584 (8)
C6A	0.7972 (9)	0.1813 (7)	0.8853 (5)	0.0666 (18)	0.584 (8)
C6B	0.8811 (14)	0.1978 (9)	0.8936 (6)	0.073 (3)	0.416 (8)
C3B	0.7530 (10)	0.4409 (9)	0.7391 (7)	0.062 (2)	0.416 (8)
C4B	0.816 (3)	0.472 (3)	0.866 (2)	0.079 (5)	0.416 (8)
C2B	0.8552 (8)	0.3244 (5)	0.7066 (4)	0.0507 (16)	0.416 (8)
C5B	0.7715 (14)	0.3116 (13)	0.9232 (7)	0.096 (4)	0.416 (8)
N1	0.47677 (19)	0.24531 (19)	0.40932 (17)	0.0468 (5)	
H2A2	0.75640	0.31040	0.61520	0.0660*	0.584 (8)
H3A1	0.79720	0.54060	0.71210	0.0750*	0.584 (8)
H2A1	0.63550	0.32370	0.69170	0.0660*	0.584 (8)
H6A1	0.68830	0.17260	0.88500	0.0800*	0.584 (8)
H6A2	0.85200	0.09700	0.92730	0.0800*	0.584 (8)
H3A2	0.94280	0.43950	0.76510	0.0750*	0.584 (8)
H4A1	0.82450	0.54210	0.92290	0.0920*	0.584 (8)
H4A2	0.67500	0.44680	0.86980	0.0920*	0.584 (8)
H5A1	0.84560	0.32270	1.02230	0.0750*	0.584 (8)
H5A2	0.97240	0.32560	0.95090	0.0750*	0.584 (8)
H2B1	0.96390	0.34930	0.73690	0.0610*	0.416 (8)
H2B2	0.83420	0.31450	0.62300	0.0610*	0.416 (8)
H3B1	0.75430	0.53050	0.69410	0.0740*	0.416 (8)
H3B2	0.64680	0.40540	0.72460	0.0740*	0.416 (8)
H4B1	0.76550	0.55630	0.89120	0.0950*	0.416 (8)
H4B2	0.92780	0.48860	0.88470	0.0950*	0.416 (8)
H5B1	0.78720	0.32050	1.00650	0.1150*	0.416 (8)
H5B2	0.66410	0.28440	0.88930	0.1150*	0.416 (8)
H6B1	0.87760	0.10500	0.93420	0.0870*	0.416 (8)
H6B2	0.98710	0.23420	0.91150	0.0870*	0.416 (8)
H1	0.522 (3)	0.290 (3)	0.372 (2)	0.068 (8)*	
H2	0.542 (3)	0.184 (3)	0.4556 (19)	0.055 (6)*	
H3	0.448 (3)	0.308 (3)	0.463 (2)	0.081 (8)*	
H4	0.393 (4)	0.201 (3)	0.365 (3)	0.090 (9)*	

### Atomic displacement parameters (Å^2^)

**Table:**

	*U*^11^	*U*^22^	*U*^33^	*U*^12^	*U*^13^	*U*^23^
S1	0.0487 (2)	0.0370 (2)	0.0367 (2)	0.0034 (2)	0.0126 (2)	0.0006 (1)
S2	0.0722 (3)	0.0415 (2)	0.0449 (2)	−0.0032 (2)	0.0132 (2)	0.0091 (2)
N2	0.1274 (16)	0.0387 (8)	0.0397 (7)	0.0076 (9)	0.0302 (9)	−0.0007 (6)
C1	0.0447 (8)	0.0371 (7)	0.0379 (7)	−0.0001 (6)	0.0150 (6)	0.0017 (6)
C2A	0.071 (3)	0.0412 (16)	0.0505 (17)	0.0079 (16)	0.0113 (16)	−0.0034 (12)
C3A	0.077 (3)	0.0394 (17)	0.068 (3)	−0.001 (2)	0.012 (3)	−0.0056 (16)
C4A	0.105 (6)	0.067 (8)	0.063 (5)	0.019 (6)	0.030 (4)	−0.021 (4)
C5A	0.071 (3)	0.072 (3)	0.0455 (18)	0.000 (2)	0.014 (2)	−0.0168 (17)
C6A	0.101 (4)	0.066 (3)	0.0404 (19)	−0.004 (3)	0.032 (3)	−0.0098 (17)
C6B	0.122 (7)	0.059 (3)	0.039 (3)	−0.011 (5)	0.024 (4)	0.000 (2)
C3B	0.058 (4)	0.059 (3)	0.067 (4)	0.016 (3)	0.014 (3)	−0.011 (3)
C4B	0.130 (11)	0.050 (4)	0.067 (7)	−0.008 (6)	0.043 (6)	−0.017 (4)
C2B	0.070 (4)	0.0338 (19)	0.052 (2)	0.007 (2)	0.022 (2)	0.0016 (16)
C5B	0.097 (6)	0.144 (9)	0.058 (4)	−0.023 (6)	0.040 (4)	−0.038 (5)
N1	0.0396 (8)	0.0394 (7)	0.0623 (9)	−0.0031 (6)	0.0141 (7)	0.0027 (7)

### Geometric parameters (Å, °)

**Table:**

S1—C1	1.7319 (17)	C2A—H2A2	0.9700
S2—C1	1.7050 (16)	C2B—H2B1	0.9700
N2—C1	1.331 (2)	C2B—H2B2	0.9700
N2—C2A	1.512 (4)	C3A—H3A1	0.9700
N2—C6A	1.515 (6)	C3A—H3A2	0.9700
N2—C2B	1.540 (5)	C3B—H3B1	0.9700
N2—C6B	1.542 (7)	C3B—H3B2	0.9700
N1—H4	0.89 (3)	C4A—H4A1	0.9700
N1—H3	0.93 (3)	C4A—H4A2	0.9700
N1—H1	0.78 (3)	C4B—H4B2	0.9700
N1—H2	0.89 (3)	C4B—H4B1	0.9700
C2A—C3A	1.515 (7)	C5A—H5A1	0.9700
C2B—C3B	1.499 (10)	C5A—H5A2	0.9700
C3A—C4A	1.639 (18)	C5B—H5B1	0.9700
C3B—C4B	1.50 (2)	C5B—H5B2	0.9700
C4A—C5A	1.465 (17)	C6A—H6A2	0.9700
C4B—C5B	1.69 (3)	C6A—H6A1	0.9700
C5A—C6A	1.489 (8)	C6B—H6B1	0.9700
C5B—C6B	1.514 (16)	C6B—H6B2	0.9700
C2A—H2A1	0.9700		
			
C1—N2—C2A	119.73 (19)	C4A—C3A—H3A2	111.00
C1—N2—C6A	118.6 (3)	C2A—C3A—H3A1	111.00
C1—N2—C2B	121.2 (2)	C4B—C3B—H3B2	110.00
C1—N2—C6B	122.8 (3)	C2B—C3B—H3B1	110.00
C2A—N2—C6A	112.6 (3)	C2B—C3B—H3B2	110.00
C2B—N2—C6B	105.9 (4)	C4B—C3B—H3B1	110.00
H1—N1—H2	109 (3)	H3B1—C3B—H3B2	109.00
H1—N1—H3	110 (3)	C3A—C4A—H4A1	110.00
H1—N1—H4	111 (3)	C3A—C4A—H4A2	110.00
H2—N1—H3	102 (2)	C5A—C4A—H4A2	110.00
H2—N1—H4	114 (3)	H4A1—C4A—H4A2	109.00
H3—N1—H4	110 (3)	C5A—C4A—H4A1	110.00
S2—C1—N2	120.70 (13)	C5B—C4B—H4B1	112.00
S1—C1—S2	118.40 (9)	H4B1—C4B—H4B2	110.00
S1—C1—N2	120.91 (13)	C3B—C4B—H4B2	111.00
N2—C2A—C3A	107.5 (3)	C5B—C4B—H4B2	112.00
N2—C2B—C3B	105.0 (5)	C3B—C4B—H4B1	112.00
C2A—C3A—C4A	103.6 (7)	C6A—C5A—H5A2	109.00
C2B—C3B—C4B	106.9 (12)	C4A—C5A—H5A1	109.00
C3A—C4A—C5A	106.5 (10)	C4A—C5A—H5A2	109.00
C3B—C4B—C5B	100.4 (15)	C6A—C5A—H5A1	109.00
C4A—C5A—C6A	111.3 (8)	H5A1—C5A—H5A2	108.00
C4B—C5B—C6B	104.9 (12)	C4B—C5B—H5B2	111.00
N2—C6A—C5A	110.3 (5)	C6B—C5B—H5B1	111.00
N2—C6B—C5B	101.5 (7)	C4B—C5B—H5B1	111.00
C3A—C2A—H2A2	110.00	H5B1—C5B—H5B2	109.00
N2—C2A—H2A1	110.00	C6B—C5B—H5B2	111.00
N2—C2A—H2A2	110.00	N2—C6A—H6A2	110.00
C3A—C2A—H2A1	110.00	C5A—C6A—H6A1	110.00
H2A1—C2A—H2A2	108.00	H6A1—C6A—H6A2	108.00
N2—C2B—H2B1	111.00	C5A—C6A—H6A2	110.00
N2—C2B—H2B2	111.00	N2—C6A—H6A1	110.00
C3B—C2B—H2B2	111.00	H6B1—C6B—H6B2	109.00
H2B1—C2B—H2B2	109.00	N2—C6B—H6B1	111.00
C3B—C2B—H2B1	111.00	N2—C6B—H6B2	112.00
C4A—C3A—H3A1	111.00	C5B—C6B—H6B1	111.00
H3A1—C3A—H3A2	109.00	C5B—C6B—H6B2	111.00
C2A—C3A—H3A2	111.00		
			
C2A—N2—C1—S1	17.9 (4)	C1—N2—C6A—C5A	159.2 (4)
C2A—N2—C1—S2	−161.9 (3)	C2A—N2—C6A—C5A	−53.7 (6)
C6A—N2—C1—S1	162.6 (4)	N2—C2A—C3A—C4A	−65.1 (8)
C6A—N2—C1—S2	−17.3 (4)	C2A—C3A—C4A—C5A	68.2 (10)
C1—N2—C2A—C3A	−152.3 (4)	C3A—C4A—C5A—C6A	−64.0 (11)
C6A—N2—C2A—C3A	61.1 (6)	C4A—C5A—C6A—N2	56.6 (10)

### Hydrogen-bond geometry (Å, °)

**Table:**

*D*—H···*A*	*D*—H	H···*A*	*D*···*A*	*D*—H···*A*
N1—H1···S2^i^	0.78 (3)	2.64 (3)	3.4029 (19)	167 (2)
N1—H2···S1	0.89 (3)	2.49 (3)	3.3565 (19)	164 (2)
N1—H3···S1^ii^	0.93 (3)	2.51 (3)	3.3967 (19)	159 (2)
N1—H4···S2^iii^	0.89 (3)	2.48 (3)	3.3632 (19)	170 (3)

Symmetry codes: (i) −*x*+3/2, *y*+1/2, −*z*+1; (ii) *x*−1/2, −*y*+1/2, *z*; (iii) −*x*+1, −*y*, −*z*+1.
